# Skin autofluorescence as a novel predictor of acute kidney injury after liver resection

**DOI:** 10.1186/s12957-021-02394-0

**Published:** 2021-09-15

**Authors:** Maciej Krasnodębski, Karolina Grąt, Marcin Morawski, Jan Borkowski, Piotr Krawczyk, Andriy Zhylko, Michał Skalski, Piotr Kalinowski, Krzysztof Zieniewicz, Michał Grąt

**Affiliations:** 1 Department of General, Transplant and Liver Surgery, Medical University of Warsaw, Warsaw, Poland; 2 Second Department of Clinical Radiology, Medical University of Warsaw, Warsaw, Poland

**Keywords:** Skin autofluorescence, Acute kidney injury, Liver resection

## Abstract

**Abstract:**

**Background:**

Skin autofluorescence (SAF) reflects accumulation of advanced glycation end-products (AGEs). The aim of this study was to evaluate predictive usefulness of SAF measurement in prediction of acute kidney injury (AKI) after liver resection.

**Methods:**

This prospective observational study included 130 patients undergoing liver resection. The primary outcome measure was AKI. SAF was measured preoperatively and expressed in arbitrary units (AU).

**Results:**

AKI was observed in 32 of 130 patients (24.6%). SAF independently predicted AKI (*p* = 0.047), along with extent of resection (*p* = 0.019) and operative time (*p* = 0.046). Optimal cut-off for SAF in prediction of AKI was 2.7 AU (area under the curve [AUC] 0.611), with AKI rates of 38.7% and 20.2% in patients with high and low SAF, respectively (*p* = 0.037). Score based on 3 independent predictors (SAF, extent of resection, and operative time) well stratified the risk of AKI (AUC 0.756), with positive and negative predictive values of 59.3% and 84.0%, respectively. In particular, SAF predicted AKI in patients undergoing major and prolonged resections (*p* = 0.010, AUC 0.733) with positive and negative predictive values of 81.8%, and 62.5%, respectively.

**Conclusions:**

AGEs accumulation negatively affects renal function in patients undergoing liver resection. SAF measurement may be used to predict AKI after liver resection, particularly in high-risk patients.

## Background

Skin autofluorescence (SAF) is an indirect measure of systemic accumulation of advanced glycation end products (AGEs). Proteins, lipids, and nucleic acids form AGEs throughout the lifetime in the presence of hyperglycemia through non-enzymatic Maillard reaction [1]. Alternatively, AGEs may be created as a consequence of reactions with particles formed under oxidative stress or are delivered to the human body with food or tobacco smoke. Therefore, age-related accumulation of AGEs is often considered as a marker of “cumulative metabolic stress” or “metabolic memory” [1, 2].

Wide spectrum of AGEs includes both fluorescent particles, such as pentosidine and pentodilysine, and non-fluorescent particles, such as *N*^ε^-carboxymethyl-lysine and *N*^ε^-carboxyethyl-lysine [3, 4]. Although SAF measurement is based on the amount of fluorescent AGEs, it is also correlated to non-fluorescent AGE content [5]. Increased accumulation of AGEs was found to be associated with atherosclerosis and vascular dysfunction, cardiovascular morbidity and mortality, peripheral artery disease, diabetes and related complications, and in particular, chronic kidney disease [5–18]. Pathogenic effects of AGEs comprise both cross-linking of other proteins with subsequent damage of extracellular matrix and activation of receptor-dependent downstream inflammatory cascades involving nuclear factor kappa-B [3, 4, 16]. In kidneys, AGE-related organ injury is largely driven by accumulation of methylgloxal, a process referred to as dicarbonyl stress [19]. Accumulating AGEs induce podocyte injury, oxidative damage, and angiotensinogen overexpression [20–22]. Those pathogenetic mechanisms may also promote acute kidney injury (AKI) in susceptible patients, yet whether AGE accumulation is associated with increased risk of developing AKI in the postoperative period after major abdominal operations remains unknown.

AKI occurs commonly in patients undergoing liver resection with an estimated incidence of 8.2–53.8%, as opposed to a 6–13.4% rate in patients after major abdominal surgery in general [23–29]. It is associated with increased morbidity and mortality rates [23, 24]. Prolonged operative time, extent of liver resection, preoperative kidney function, and patient age were previously commonly identified as important risk factors [23, 24, 28]. The standard definition of AKI is based on the Kidney Disease Improving Global Outcomes (KDIGO) criteria including serum creatinine increase by 0.3 mg/dL over 48 h or 50% over 7 days or urine output < 0.5 mL/kg/h for 6 h [30]. However, lack of clinical relevance of isolated oliguria without creatinine increase was recently confirmed in liver resection studies [25, 27]. Creatinine-only criteria for defining AKI were also approved outside the clinical care setting in a 2020 report from Improving Global Outcomes (KDIGO) Consensus Conference [31].

The primary hypothesis for this study was that increased accumulation of AGEs is a risk factor for developing AKI in patients undergoing liver resection. The aim was to evaluate whether SAF measurement may be used for prediction of AKI after liver resection.

## Methods

This was a prospective observational study performed in the Department of General, Transplant and Liver Surgery of the Medical University of Warsaw between December 2018 and November 2019. Inclusion criteria comprised open liver resection for suspected malignancy, age between 18 and 70 years, and provision of informed consent. Patients with skin phototypes IV–VI in Fitzpatrick’s classification, and those with body mass index (BMI) > 35 kg/m^2^ were excluded. The study protocol was approved by the institutional review board of the Medical University of Warsaw. All participants provided informed consent before inclusion in the study.

The primary outcome measure was AKI within 7 postoperative days. It was defined according to KDIGO criteria as either increase in serum creatinine concentration by at least 0.3 mg/dL over 48 h or by at least 50% over 7 days. Stage I, II, and III AKI were further defined as a serum creatinine concentration increase by 50–99%, 100–199%, and over 200%, respectively. Stage II and III AKI was considered severe [30]. Urine output was not considered for the purpose of diagnosing AKI.

SAF was the primary factor of interest. It was measured using AGE Reader (Diagnoptics Technologies B.V., Groningen, The Netherlands) based on a system of photodiodes. Following illumination of approximately 4 cm^2^ of skin with light wavelength between 300 and 420 nm with peak intensity at 370 nm, the light emitted and reflected by the skin was assessed. SAF value was expressed in arbitrary units (AU), expressing the ratio of average intensity of emitted light with wavelength of 420–600 nm to average intensity of reflected light with the wavelength of 300–420 nm, multiplied by 100. Measurements were done in the immediate preoperative period on the anterior side of the forearm, approximately 10 cm below the elbow fold. Average value from 3 separate assessments was included in the analyses.

Liver resections were performed through bilateral subcostal incisions. Parenchymal transection was done with either ultrasonic or waterjet device. Pringle maneuver was applied selectively. Major resections were defined as removal of more than 2 Couinaud segments. Postoperatively, patients were transferred to surgical intensive care unit for at least 24 h. Serum creatinine concentration was routinely assessed preoperatively and postoperatively at the day of operation, day 1, day 3 or 4, and in subsequent days, when clinically indicated. Severe morbidity was defined as development of complication of grades III-V in the Clavien-Dindo classification [32].

Quantitative and qualitative variables were presented as medians with interquartile ranges and as numbers with frequencies, respectively. Chi-square test and Mann-Whitney *U* test were used for intergroup comparisons of qualitative and quantitative variables. Logistic regression was used for univariable and multivariable analyses of risk factors for AKI. Preoperative creatinine concentration and factors significantly associated with AKI were included in the multivariable model. Receiver operating characteristics (ROC) analyses were performed to choose optimal cut-offs of quantitative variables in prediction of AKI, basing on the highest Youden index. Odds ratios (ORs) and areas under the curve (AUCs) were presented with 95% confidence intervals. The level of significance was set at 0.05. STATISTICA version 13 (TIBCO Software Inc., Palo Alto, USA) software was used for computing statistical analyses.

## Results

A total of 130 patients were included in the study. Baseline characteristics of the study cohort are presented in Table 1. Any-stage AKI was diagnosed in 32 patients (24.6%), including 9 with severe AKI (6.9%). In particular, stage I, stage II, and stage III AKI occurred in 23, 8, and 1 patient, respectively. None of them required renal replacement therapy in the 7-day postoperative period. Severe morbidity rate was 8.5% (defined as development of complication of grades III–V in the Clavien-Dindo classification) [32]. The length of stay did not differ between patients without and with AKI (median 7 vs. 7.5 days, *p* = 0.063), but was significantly longer for severe AKI (median 7 vs. 11 days, *p* = 0.008). One patient died in the postoperative period in the course of massive pneumonia.

**Table 1:** Baseline characteristics of 130 patients included in the study cohort

	Median (IQR) or n (%)
Patient sex	
male	67 (51.5%)
female	63 (48.5%)
Patient age (years)	60 (51-65)
Skin autofluorescence (AU)	2.3 (1.9-2.6)
Body mass index (kg/m^2^)	26.7 (23.9-29.7)
Diabetes	16 (12.3%)
Arterial hypertension	44 (33.8%)
Preoperative laboratory tests	
white blood count (10^3^/mm^3^)	6.1 (5.3-7.7)
hemoglobin (g/dL)	13.4 (12.7-14.1)
platelets (10^3^/mm^3^)	223 (190-263)
creatinine (mg/dL)	0.8 (0.7-1.0)
bilirubin (mg/dL)	0.5 (0.4-0.7)
INR	1.0 (1.0-1.1)
Primary diagnosis	
colorectal liver metastases	54 (41.5%)
non-colorectal liver metastases	15 (11.5%)
hepatocellular cancer	14 (10.8%)
gallbladder cancer	14 (10.8%)
intrahepatic cholangiocarcinoma	11 (8.5%)
extrahepatic cholangiocarcinoma	3 (2.3%)
other	19 (14.6%)
Extent of liver resection	
minor	74 (56.9%)
major	56 (43.1%)
Operative time (hours)	3.5 (2.8-4.3)

IQR interquartile range, AU arbitrary unit, INR international normalized ratio

**Fig. 1:**
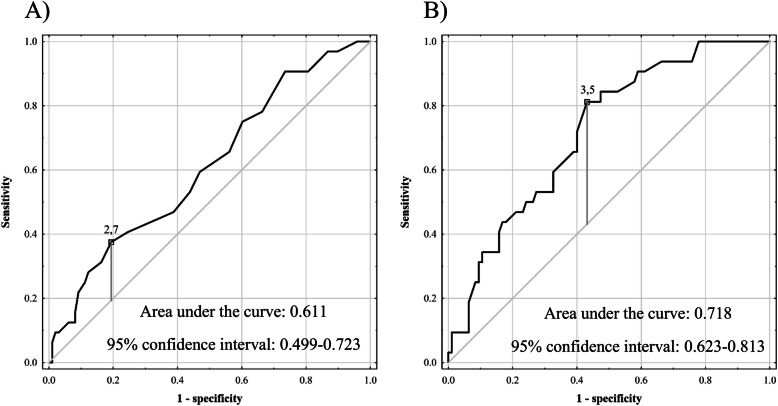
Receiver operating characteristics curves for prediction of acute kidney injury after liver resection based on **A** skin autofluorescence and **B** operative time

SAF was a significant predictor of AKI on univariable analysis (*p* = 0.039; Table 2). Other risk factors comprised operative time (*p* = 0.001), male sex (*p* = 0.010), and major liver resection (*p* < 0.001). SAF was independently associated with the development of AKI (*p* = 0.047), in addition to the extent of liver resection (*p* = 0.019) and operative time (*p* = 0.046) in a multivariable model including those 3 variables, patient sex, and preoperative creatinine concentration. The optimal cut-offs for SAF and operative time in prediction of AKI were 2.7 AU and 3.5 h, respectively, with the corresponding AUCs of 0.611 (95% CI 0.499–0.723; Fig. 1A) and 0.718 (95% CI 0.623–0.813; Fig. 1B). Sensitivity, specificity, PPV, and NPV were 37.5%, 80.6%, 38.7%, and 79.8%, respectively, for the established SAF cut-off, and 81.3%, 56.8%, 38.8%, and 90.0%, respectively, for the established operative time cut-off. The rate of AKI in patients with SAF ≥ 2.7 AU was 38.7% (12 of 31) as compared to 20.2% (20 of 99) in patients with SAF < 2.7 AU (*p* = 0.037).

**Table 2:** Analyses of risk factors for the occurrence of acute kidney injury after liver resection

Factors	Univariable		Multivariable	
	OR (95% CI)	p	OR (95% CI)	p
Male sex	1.75 (1.14-2.67)	.010	1.35 (0.81-2.27)	.252
Patient age	1.03 (0.99-1.08)	.110		
Skin autofluorescence	1.98 (1.03-3.80)	.039	2.16 (1.01-4.62)	.047
Body mass index	1.02 (0.93-1.12)	.729		
Diabetes	1.66 (0.97-2.86)	.065		
Arterial hypertension	1.22 (0.81-1.84)	.352		
Preoperative creatinine	2.72 (0.58-12.87)	.206	0.61 (0.08-4.31)	.616
Major liver resection	2.49 (1.58-3.91)	<.001	1.83 (1.10-3.05)	.019
Operative time	1.67 (1.23-2.26)	.001	1.43 (1.01-2.04)	.046

OR – odds ratio; 95% CI - 95% confidence interval. Odds ratios were calculated per: 1 year increase for patient age; 1 arbitrary unit increase for skin autofluorescence; 1 kg/m^2^ increase for body mass index; 1 mg/dL increase for preoperative creatinine; 1 hour increase for operative time

**Fig. 2:**
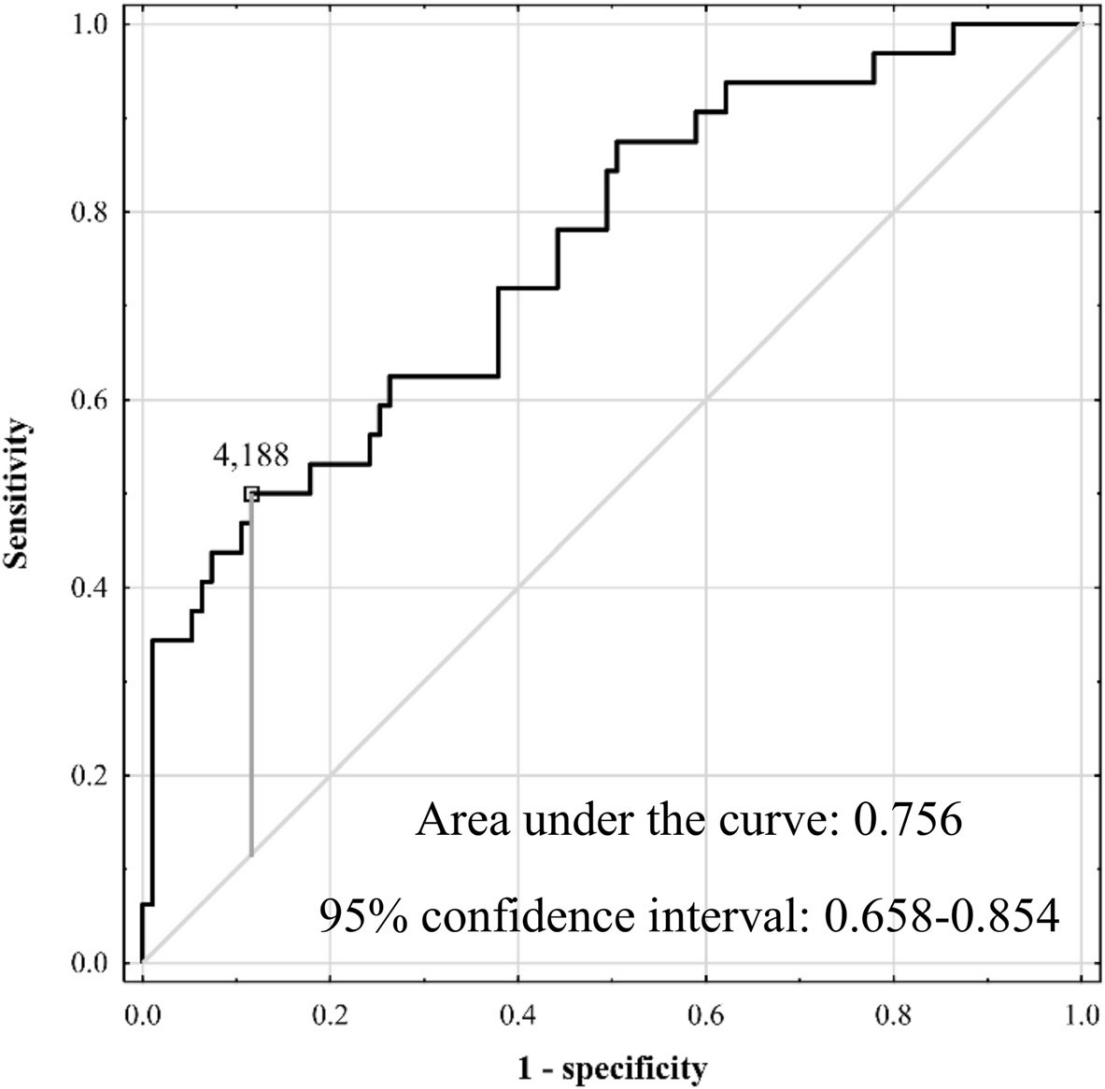
Receiver operating characteristics curve for prediction of acute kidney injury after liver resection based on a risk score utilizing skin autofluorescence, extent of resection, and operative time

A risk score for AKI basing on SAF, operative time, and extent of liver resection ([0.771 × SAF] + [0.359 × operative time in hours] + 0.607 if major resection) was associated with AUC of 0.756 (95% CI 0.658–0.854). Sensitivity, specificity, PPV, and NPV rates were 50.0%, 88.4%, 59.3%, and 84.0%, respectively, for a risk score cut-off of 4.188 (Fig. 2). AKI occurred in 59.3% (16 of 27) of patients with risk score value ≥ 4.188 as compared to 16.0% (16 of 100) of patients with lower values (*p* < 0.001).

**Fig. 3:**
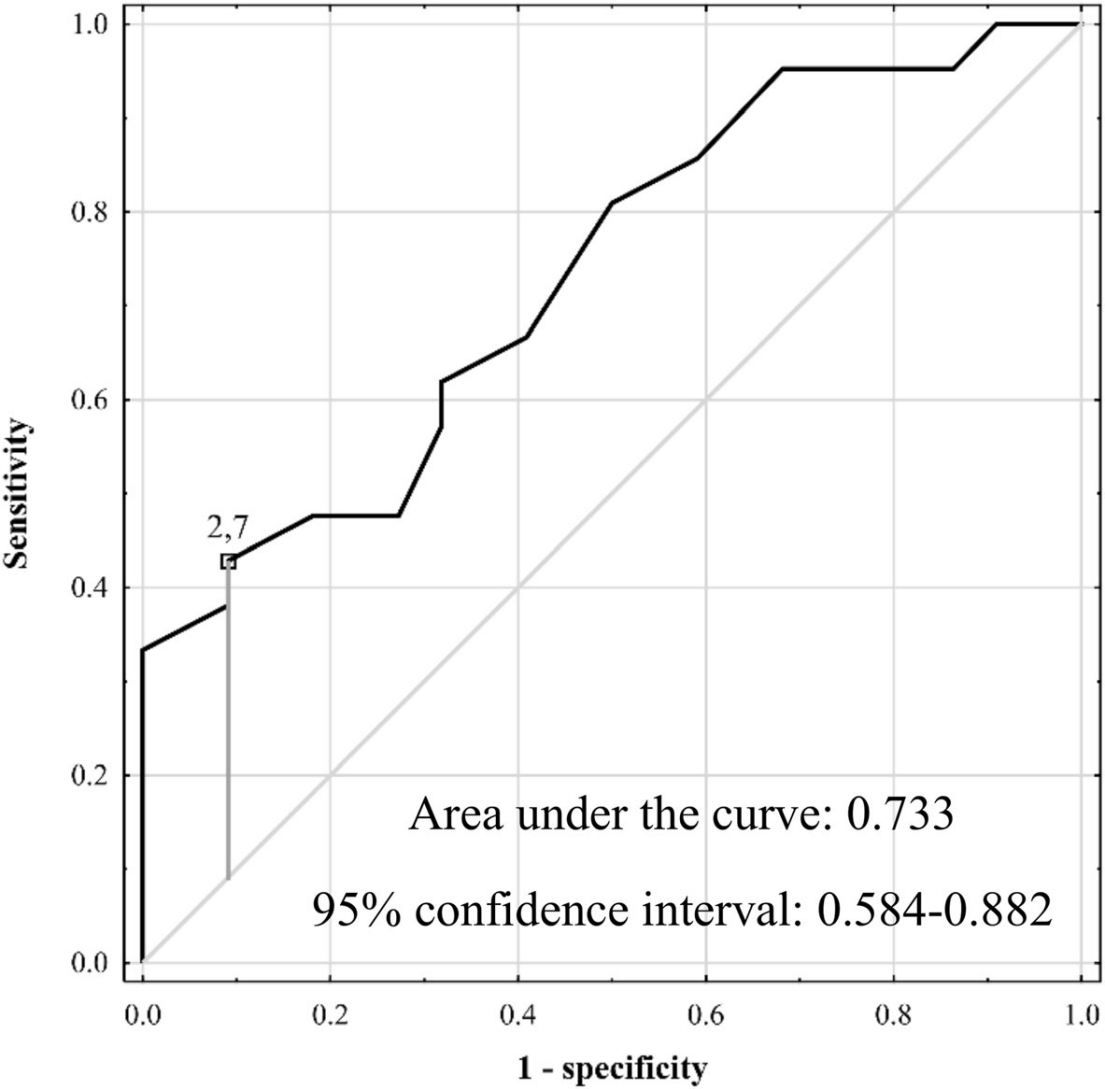
Receiver operating characteristics curves for prediction of acute kidney injury after liver resection based on skin autofluorescence in a subgroup of patients undergoing major resections lasting ≥ 3.5 h

Regardless of SAF, AKI occurred in 6.4% (3 of 47) of patients after minor resections lasting < 3.5 h, in 20.8% (5 of 24) of patients undergoing minor resections lasting ≥ 3.5 h, in 23.1% (3 of 13) of patients after major resections lasting < 3.5 h, and in 48.8% (21 of 43) of patients undergoing major resections lasting ≥ 3.5 h (*p* < 0.001). SAF was a significant risk factor for developing AKI in the high-risk group of patients undergoing major resections lasting ≥ 3.5 h (*p* = 0.010; Table 3). The optimal cut-off for SAF in prediction of AKI in this high-risk subgroup was also 2.7 AU with AUC of 0.733 (95% CI 0.584–0.882) and sensitivity, specificity, PPV, and NPV rates of 42.9%, 90.9%, 81.8%, and 62.5%, respectively (Fig. 3). In subgroup analysis of patients undergoing major resections lasting ≥ 3.5 h, the rate of AKI was 81.8% (9 of 11) in case of SAF ≥ 2.7 AU as compared to 37.5% (12 of 32) in case of lower SAF values (*p* = 0.011).

**Table 3:** Subgroup analyses of the associations between skin autofluorescence and occurrence of acute kidney injury after liver resection

Subgroup	Factor	OR (95% CI)	p
Major resections lasting ≥3.5 hours	SAF	6.69 (1.57-28.58)	.010
Major resections lasting <3.5 hours or minor resections lasting ≥3.5 hours	SAF	0.48 (0.11-2.09)	.329
Minor resections lasting <3.5 hours	SAF	-^a^	-^a^

^a^ – not calculated due to only 3 events in the subgroup. OR – odds ratio; 95% CI – 95% confidence interval; SAF – skin autofluorescence. Odds ratios were calculated per 1 arbitrary unit increase

In contrast to any-stage AKI, the association between SAF and development of severe AKI was not significant (OR 2.19 per 1 AU increase; 95% CI 0.80–5.99; *p* = 0.126).

## Discussion

Although the pathogenic role of systemic accumulation of AGEs in development and progression of chronic kidney disease is being intensively studied, there was no available data regarding development of AKI in postoperative setting [1, 3, 18, 19]. Only a single previous report pointed towards higher SAF values in patients admitted for AKI in general [33]. This study provides first data on the negative effects of AGEs accumulation on development of AKI in patients undergoing liver resection. The present results indicate that SAF, as an indirect measure of AGEs skin content, may be applied for preoperative prediction of AKI.

Considering all patients undergoing liver resection, the association between SAF and development of AKI was significant, yet the predictive performance of SAF was limited as reflected by low AUC, low sensitivity, and moderate specificity. Nevertheless, the use of SAF cut-off of 2.7 AU points towards identification of patients at nearly doubled risk of developing AKI in the postoperative period. Further, PPV of nearly 40% observed for the established SAF cut-off is remarkably better than the 25% PPV related to intraoperative oliguria in major abdominal surgery [34]. Consistent with other reports, extent of liver resection and duration of surgical procedure were the two main determinants of AKI occurrence in patients undergoing liver resection [23–25, 28, 35]. As preoperative serum creatinine concentration was previously commonly identified as important predictor of AKI, it was included in the multivariable model despite a lack of significance on univariable analysis [28, 36]. Importantly, the association between SAF and occurrence of AKI remained significant following adjustment for the confounding effects of extent of liver resection and its duration, preoperative kidney function, and patient sex.

While the prediction of AKI based only on SAF was suboptimal and useful for general stratification of patients into low- and high-risk group, a risk score based on SAF, extent of resection and operative time seems to enable more accurate prediction. Assuming precise preoperative planning and estimation of operative time, application of the simple 3-factor score may lead to preoperative prediction of half of all AKI cases with nearly 6 of 10 patients with positive prediction developing AKI. Notably, despite its simplicity due to inclusion of only 3 variables, an AUC of 0.756 seems favorable compared to the corresponding values reported for far more complex models. In a study by Slankamenac et al., a model based on 7 preoperative variables, namely cardiovascular disease, chronic renal failure, diabetes, serum alanine transaminase activity and bilirubin concentration, age, and sex was associated with an AUC of 0.80 [37]. Further studies by the same group revealed similar AUC of 0.81 for combination of that preoperative score with intraoperative parameters [38]. Regarding prediction of AKI after major gastrointestinal surgery in general, a 6-variable model provided a c-statistic of 0.66 [36]. Notably, AUCs for algorithms based on machine learning were in the range of 0.628–0.772 [39]. On the contrary, an AUC of nearly 0.90 was found for urine neutrophil gelatinase-associated lipocalin (NGAL) concentration assessed 12 h postoperatively, yet these results seem to be related to early detection of AKI rather than prediction of its development [40]. Considering that urine NGAL measurement is not performed routinely despite its high accuracy, the results of the present study point towards measuring NGAL for early detection of AKI in patients with high risk score values.

Potential advantages in clinical application of SAF measurement in prediction of AKI in patients after liver resection are clearly visible in patients at otherwise the highest risk of its development. While prolonged excessive liver resections are obviously associated with the highest risk of developing AKI, SAF efficiently stratifies patients into high and almost ultimate risk of recurrence. Accordingly, the risk of AKI was below 10% in patients after minor resections not exceeding 3.5 h, approximately 20% in those after minor resections exceeding 3.5 h and after major resections not exceeding 3.5 h, and nearly 50% in those undergoing major resections lasting over 3.5 h. Importantly, the SAF cut-off value of 2.7 AU well-stratified the patients into those with approximately 80% and 40% risk of AKI. From a clinical point of view, SAF measurement at the level of or exceeding 2.7 AU may be considered as indicating AKI in the postoperative period after major long-lasting liver resections.

The general incidence of AKI reported in the present study is relatively high, considering previous relevant reports [23–25, 28, 41, 42]. However, in contrast to previous studies, the present analysis was based on a prospective observational study, potentially increasing its sensitivity regarding AKI detection in the absence of clinical symptoms. This is supported by the similarity in cumulative incidence of stage II and stage III AKI, referred to as severe, between the present and previous reports [23, 24]. Notably, while no significant association was found between SAF and development of severe AKI, the incidence was more than 3-fold higher in patients with SAF equal to or exceeding 2.3 AU. Whether a lack of statistical significance is due to a lack of true association or due to lack of power remains an open question, yet the present study is obviously underpowered to detect differences in severe AKI rates dependent upon SAF values.

The present study is subject to several limitations. First, accumulation of AGEs was measured indirectly by assessment of SAF and does not provide any data regarding particular AGEs relevant in the pathogenesis of postoperative AKI. Second, estimation of AGE content basing on SAF measurement may be confounded by the presence of other skin fluorophores, such as nicotinamide adenine dinucleotide [43]. Nevertheless, SAF was previously validated as a relatively accurate measure of systemic accumulation of AGEs and SAF variance is primarily dependent upon AGE content [5, 43]. However, the potential casual/random relationship of SAF and AKI in the postoperative period cannot be underestimated as well. The AKI can occur due to multifactorial confounders that were not assessed like hypovolemia, sepsis, intravenous contrast administrations, and/or drug interactions. There was no routine protocol for performance of biochemical measurements of serum creatinine concentration beyond day 4, yet the potential underestimation of AKI incidence in days 5–7 is highly unlikely due to 24.6% of patients diagnosed with AKI. Finally, vast majority of patients with AKI were diagnosed as stage I and the study was underpowered to detect significant association between SAF and development of severe AKI. However, considering the negative effects of SAF on developing AKI in general and available data on the pathogenetic background of AGEs accumulation in kidney injury, a consistent effect on each stage of AKI may be hypothesized, yet this remains to be elucidated.

## Conclusions

In conclusion, this is the first study to provide evidence for significant role of AGEs accumulation in development of AKI in patients undergoing major abdominal surgery. Measurement of SAF should be performed in patients undergoing liver resection in order to aid preoperative prediction of AKI, particularly in most susceptible patients undergoing major and prolonged surgical procedures.

## Data Availability

The datasets generated during and/or analyzed during the current study are available from the corresponding author on reasonable request.
